# CXCL14 Preferentially Synergizes With Homeostatic Chemokine Receptor Systems

**DOI:** 10.3389/fimmu.2020.561404

**Published:** 2020-10-05

**Authors:** Ariadni Kouzeli, Paul J. Collins, Mieke Metzemaekers, Max Meyrath, Martyna Szpakowska, Marc Artinger, Sofie Struyf, Paul Proost, Andy Chevigne, Daniel F. Legler, Matthias Eberl, Bernhard Moser

**Affiliations:** ^1^Division of Infection and Immunity, Cardiff University School of Medicine, Cardiff, United Kingdom; ^2^Institute of Immunology and Immunotherapy, University of Birmingham, Birmingham, United Kingdom; ^3^Laboratory of Molecular Immunology, Rega Institute for Medical Research, KU Leuven, Leuven, Belgium; ^4^Department of Infection and Immunity, Immuno-Pharmacology and Interactomics, Luxembourg Institute of Health (LIH), Esch-sur-Alzette, Luxembourg; ^5^Biotechnology Institute Thurgau (BITg), University of Konstanz, Kreuzlingen, Switzerland

**Keywords:** chemokines, signal transduction, synergism, migration, cell localization, CXCR4, CXCR5, CCR7

## Abstract

Reflecting their importance in immunity, the activity of chemokines is regulated on several levels, including tissue and context-specific expression and availability of their cognate receptor on target cells. Chemokine synergism, affecting both chemokine and chemokine receptor function, has emerged as an additional control mechanism. We previously demonstrated that CXCL14 is a positive allosteric modulator of CXCR4 in its ability to synergize with CXCL12 in diverse cellular responses. Here, we have extended our study to additional homeostatic, as well as a selection of inflammatory chemokine systems. We report that CXCL14 strongly synergizes with low (sub-active) concentrations of CXCL13 and CCL19/CCL21 in *in vitro* chemotaxis with immune cells expressing the corresponding receptors CXCR5 and CCR7, respectively. CXCL14 by itself was inactive, not only on cells expressing CXCR5 or CCR7 but also on cells expressing any other known conventional or atypical chemokine receptor, as assessed by chemotaxis and/or β-arrestin recruitment assays. Furthermore, synergistic migration responses between CXCL14 and inflammatory chemokines CXCL10/CXCL11 and CCL5, targeting CXCR3 and CCR5, respectively, were marginal and occasional synergistic Ca^2+^ flux responses were observed. CXCL14 bound to 300-19 cells and interfered with CCL19 binding to CCR7-expressing cells, suggesting that these cellular interactions contributed to the reported CXCL14-mediated synergistic activities. We propose a model whereby tissue-expressed CXCL14 contributes to cell localization under steady-state conditions at sites with prominent expression of homeostatic chemokines.

## Introduction

CXCL14 is one of the least understood chemokines, featuring remarkable amino acid sequence conservation across a wide range of species and constitutive expression in border-lining tissues by epithelial cells, fibroblasts and macrophages (1). The study of target cells for CXCL14, and hence its role in physiological and pathophysiological processes, has been hampered by the lack of identification of its receptor. Nevertheless, in accordance with the definition of chemokines, CXCL14 was shown to be a chemoattractant for a subset of human immune cells, including blood monocytes, immature DCs and NK cells but not lymphocytes, which do not migrate in response to CXCL14 (2–6). Induction of Ca^2+^ mobilization and sensitivity to *Bordetella pertussis* toxin treatment in monocytes suggests that the elusive CXCL14 receptor is a prototypical chemokine receptor that requires G_α i_-type G-proteins for signaling (3). CXCL14 does not belong to the subset of chemokines whose expression is induced only under inflammatory conditions. In fact, inflammatory stimuli (LPS, TNF-α, ROS) and growth factors (VEGF, EGF) that signal through the MEK/ERK pathway were shown to inhibit the constitutive expression of CXCL14 (7–10), an effect frequently linked with epigenetic silencing (8, 11–14). Positive modulators of CXCL14 expression are less clear and vary depending on the cellular context (15–17).

Mutant mice lacking CXCL14 do not show aberrant numbers or tissue distribution of immune cells but suffer from developmental and/or metabolic defects, as evidenced by decreased survival rate of neonates, reduced body weight of adults and defects in glucose metabolism (18–21). These findings suggest subtle involvement of CXCL14 in steady-state processes that may go beyond the control of immunity. A large body of literature discusses the pro-and anti-tumor effects of CXCL14 [reviewed in Yang et al. (22)]. The tumor-suppressor activity of CXCL14 is more widely documented, with loss of CXCL14 expression by epigenetic silencing coinciding with tumor progression (8, 12–14). Conversely, stromal cell-derived CXCL14 was frequently shown to promote tumor growth *via* diverse mechanisms, including epithelial to mesenchymal transition, coupled with tumor cell metastasis as well as indirect support of angiogenesis and tumor growth (22). The effect of CXCL14 on fibroblasts is noteworthy, as CXCL14 induced a fibrosis gene expression profile, linking this chemokine with lung disease (23–25), whereas stimulation of tumor-associated fibroblasts with CXCL14 led to the generation of tumor-promoting factors (26–28). Collectively, mounting evidence supports a role for CXCL14 in controlling the activity and functions of certain tissue cell types, in addition to circulating immune cells, in the context of health and disease. This warrants an extensive investigation into the relationships and interactions that occur between CXCL14 and other chemokine family members.

We have previously reported that CXCL14 acts as a positive allosteric modulator of CXCR4 during synergistic cell responses with CXCL12 (29). CXCL14 by itself was inactive yet, in combination with low CXCL12 concentrations, induced strong chemotactic responses in CXCR4-expressing cells. Synergism with CXCL14 required the presence of CXCR4 whose cell surface aggregation was promoted by CXCL14 binding. In the present study we have asked the question whether the observed CXCL14 synergism is restricted to CXCL12 and its receptor CXCR4 by examining other, CXCR4-unrelated, homeostatic and inflammatory chemokine systems. We report that CXCL14 did not activate any of the known classical or atypical chemokine receptors but exhibited synergistic responses with the homeostatic chemokines CXCL13, CCL19 and CCL21 in inducing the chemotactic migration of cells expressing the corresponding chemokine receptors CXCR5 and CCR7. We further report that synergism of CXCL14 with inflammatory chemokines was much less obvious. We discuss our novel findings on the basis of the site-specific co-expression of CXCL14 and its synergizing homeostatic chemokines CXCL12, CXCL13, CCL19 and CCL21, and discuss models of pleiotropic chemokine co-operation in epithelial tissues.

## Materials and Methods

### Chemokines and Other Reagents

Synthetic chemokines (CXCL14, CCL19, CCL21, CXCL13, CCL5, CXCL10 and CXCL11) were chemically synthezised, as previously described (30). CCL19^Dy69P1^ was produced as previously described (31, 32). Human CXCL14 conjugated to the fluorochrome Alexa Fluor 647 attached to a C-terminal lysine residue was purchased from Almac Sciences (Edinburgh, United Kingdom).

### Isolation of Primary Human Cells

All research involving work with human venous blood samples was approved by the local research ethics commission and informed consent was obtained from each participant. Peripheral blood mononuclear cells (PBMC) were isolated from the heparinized blood of healthy volunteers by centrifugation over a Lymphoprep (Axis Shield, Dundee, United Kingdom) gradient. Total CD3^+^ T cells were isolated from PBMC using a Pan-T cell isolation kit (Miltenyi Biotec, Bisley, United Kingdom), according to manufacturer’s instructions.

### *In vitro* Cell Cultures

Primary cell cultures were maintained in RPMI-1640 medium that was supplemented with 10% (v/v) foetal calf serum (FCS), 2 mM L-glutamine, 1 mM sodium pyruvate, 1% (v/v) non-essential amino acids, and 50 μg/ml penicillin/streptomycin (cRPMI; all from Thermo Fisher Scientific, Waltham, MA, United States). Murine pre-B cell line 300-19 was cultured in the same medium that was supplemented with 50 μM 2-mercaptoethanol (2-ME;Thermo Fisher Scientific) and 1.5 μg/ml puromycin for selection. Human embryonic kidney (HEK293T) cells were cultured in DMEM medium supplemented with 10% (v/v) FCS, 1% non-essential amino acids and 50 μg/ml penicillin/streptomycin. Cells were maintained in a humidified incubator at 37°C and a mixture of 95% air, 5% CO_2_. 300-19 cells have been stably transfected and routinely used by our group and others (33). Parental (untransfected) and 300-19 cells that were stably transfected with various chemokine receptors were maintained at a cell density not exceeding 2 × 10^6^ cells/ml. All cell lines were routinely tested for mycoplasma contamination by RT-PCR.

### Transwell Chemotaxis Assay

Chemokines were resuspended in chemotaxis buffer [plain RPMI-1640 containing 1% pasteurized plasma protein solution (5% PPL SRK; SwissRed Cross Laboratory, Bern, Switzerland) and 20 mM HEPES (Thermo Fisher Scientific)] to the desired concentration and 235 μl was placed in the lower chamber of Transwell 96-well plates (4.26 mm, 5.0 μm pore; Corning, St. David’s Park, United Kingdom). A well containing chemotaxis buffer with no chemokine (buffer only control) was used as a negative control. Polycarbonate filters were placed in the wells and the plate was allowed to equilibrate at 37°C. PBMC or 300-19 cells were resuspended in prewarmed chemotaxis buffer at 2 × 10^6^ cells/ml. Cells were allowed to rest for 30 min at 37°C before the assay. Cells (160,000 in 80 μl) were placed in the upper well of the Transwell and the plate was incubated at 37°C for 2–5 h. Upon termination of the assay, migrated cells were collected from the lower chamber and transferred to 96-well plates for staining. Following staining, cells were resuspended in 75 μl PBS containing 2% FCS + 0.02% sodium azide (FACS buffer). Accu-Check (25 μl) counting beads (Thermo Fisher Scientific) were added to each sample to enable accurate cell counts determined by flow cytometry. Cell migration data were expressed either by a percentage of total input cells or a migration index, which is defined as the number of cells migrated toward a chemokine divided by the number of cells migrated in response to buffer (buffer only control).

### Flow Cytometry

Single cell suspensions were incubated with Fixable Aqua Dead Cell Stain Kit (Thermo Fisher Scientific) to allow exclusion of dead cells. Following blocking of endogenous Fc receptors, cells were incubated with fluorochrome-conjugated mAbs against the following human cell-surface markers (conjugate and clone indicated in brackets): CD3 (APC-H7, SK7), CCR7 (PECy7, 3D12), CCR5 (PE, 2D7), CD4 (BV421, RPA-T4; BD Biosciences, Oxford, United Kingdom); CD19 (APC, SJ25C1, eBioscience, Hatfield, United Kingdom); CXCR3 (FITC, 498801.111; R&D, Minneapolis, MN, United States). Staining with mAbs was performed in FACS buffer for 30 min at 4°C. Isotype and Fluorescence-Minus-One controls were used as appropriate. Sample acquisition was performed by using a FACS Canto II instrument (BD Biosciences). Cell aggregates were excluded based on light scatter properties. Data was analyzed using FlowJo software (Version 10.4, TreeStar, Ashland, OR, United States).

### Ca^2+^ Mobilization Assay

Chemokine receptor-transfected pre-B 300-19 cells (10 × 10^6^ cells/ml) in cRPMI were treated with 2.5 μM Fura-2-AM (HelloBio, Bristol, United Kingdom), 0.01% (w/v) Pluronic-F127, Thermo Fisher Scientific) and 125 μM Probenecid solution (Sigma-Aldrich, Gillingham, United Kingdom) for 30 min at room temperature. Cells were washed twice with cRPMI and resuspended in pH 7.0 Ca^2+^ buffer (Hanks Balanced Salt Solution [HBSS; Invitrogen]) containing Ca^2+^ and Mg^2+^ and complemented with 10 mM HEPES (Gibco) and 0.1% (*v*/*v*) FCS enriched with 125 μM Probenecid, while being kept on ice. Cells were resuspended in Ca^2+^ buffer supplemented with Probenecid at final concentrations of 10^6^ cells/ml. A spectrophotometer (Fluorescence Spectrophotometer F-7000, Hitachi, Japan) was used to measure fluorescence and intracellular Ca^2+^ concentrations ([Ca^2+^]_i_) were calculated using the Grynkiewicz equation (34). For each individual measurement, 900,000 cells were preheated for 10 min at 30°C prior to stimulation with a chemokine. Chemokine was injected after 100 s of recording and recording continued up to 300 s. R_max_ and R_min_ values were determined by treatment of cells with 50 μM digitonin and 10 mM EGTA (Sigma-Aldrich), respectively, in 20 mM Tris (pH 8.5; Merck, Darmstadt, Germany).

### Chemokine Competition Assay

For chemokine competition assays, 2.5 × 10^5^ 300-19 pre-B cells stably expressing CCR7-eGFP in staining buffer (145 mM NaCl, 5 mM KCl, 1 mM MgCl_2_, 1 mM CaCl_2_, 1 mM sodium phosphate, 5 mM HEPES; pH 7.5) were incubated with 25 nM fluorescently labeled human CCL19 conjugated to Dy649P1 (Dyomics, Jena, Germany) in the presence of graded concentrations of unlabeled human CXCL14 or CCL19 at 37°C for 60 min. Cells were washed and analyzed on a LSRII flow cytometer (BD Biosciences, San Jose, CA, United States) recording eGFP and chemokine fluorescence. Flow cytometry data were evaluated with FlowJo V10 and displayed using GraphPad Prism 8.0 (GraphPad, San Diego, CA, United States). IC_50_ values were calculated by three parametric non-linear regression for agonist concentration over response.

### β-Arrestin Assay

β-arrestin recruitment to chemokine receptors in response to CCL19, CXCL14 or positive control chemokines was monitored by NanoLuc complementation assay [NanoBiT, Promega, Madison, WI, United States (35)] as previously described (36). In brief, 6.5 × 10^5^ HEK293T cells were plated per well in a 12-well dish (6 × 10^6^ per 10 cm dish for concentration response curves) and 24 h later co-transfected with pNBe vectors encoding a chemokine receptor C-terminally tagged to the luciferase fragment SmBiT and human β-arrestin-2 N-terminally fused to LgBiT. 24 h post-transfection cells were harvested, incubated for 25 min at 37°C with Nano-Glo Live Cell substrate diluted 200-fold and distributed into white 96-well plates (1 × 10^5^ cells per well). Ligand-induced, β-arrestin recruitment to chemokine receptors was evaluated with a Mithras LB940 luminometer (Berthold Technologies, Bad Wildbad, Germany) for 20 min. For concentration-response curves, 3 nM CCL19 was co-incubated with increasing concentrations of CXCL14 for 15 min at room temperature before treatment. For screening experiments, 200 nM of one known agonist chemokine listed in the IUPHAR repository of chemokine receptor ligands was added as positive control to each receptor.

### Statistical Analyses

All statistical analyses were performed using GraphPad Prism version 8 for macOS (GraphPad Software, Inc., San Diego, CA, United States). All data are expressed as mean values and error bars represent the SD or SEM. The D’Agostino-Pearson omnibus normality test was carried out to determine normality of the data. Friedman test followed by Dunn’s multiple comparison test, Wilcoxon matched-pairs signed rank test or Holm-Sidak test were used to assess significance of differences in paired experimental data. For all analyses, *p* values below 0.05 were considered significant and grouped according to ^∗^ = *p* < 0.05, ^∗∗^ = *p* < 0.01, ^∗∗∗^ = *p* < 0.001, ^****^ = *p* < 0.0001.

## Results

### CXCL14 Synergizes With the CCR7 Ligands CCL19 and CCL21

**FIGURE 1:**
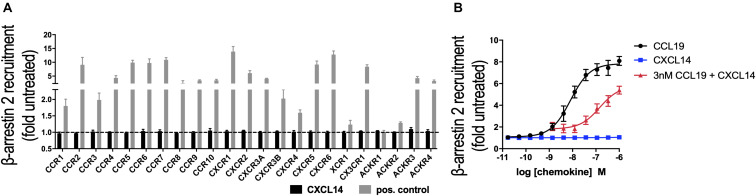
CXCL14 is not an agonist for any known chemokine or atypical chemokine receptor but synergizes with CCL19. **(A)** Agonist activity of CXCL14 (300 nM) toward 19 classical and 4 atypical chemokine receptors evaluated in a β-arrestin-2 recruitment assay. Results are expressed as fold-change over baseline and are presented as mean + SEM of three independent experiments. For all receptors, 200 nM of one known agonist chemokine listed in the IUPHAR repository of chemokine receptor ligands was added as positive control. **(B)** Agonist activity and potency of CCL19, CXCL14, or CXCL14 in presence of 3 nM CCL19 in inducing β-arrestin-2 recruitment to CCR7. CCL19 alone and CXCL14 alone were included as positive and negative control responses, respectively. Data are expressed as fold-change over non-treated cells (baseline) and are presented as means ± SEM of 3–4 independent experiments.

In its role as a positive allosteric modulator, CXCL14 synergizes with CXCL12 by direct binding to CXCR4, without inducing CXCR4-mediated signaling in the absence of CXCL12 (29). To expand our CXCL14 synergy studies to other chemokine/chemokine receptor systems, we examined all known human chemokine and atypical chemokine receptors in a global screen involving β-arrestin recruitment as a functional read-out (36, 37). β-arrestin recruitment is an early cellular response to chemokines, requires prior phosphorylation of chemokine receptors by G-protein coupled receptor (GPCR) kinases and results in receptor internalization. As shown in Figure 1A, 300 nM of synthetic or recombinant (not shown) CXCL14 did not induce β-arrestin-2 recruitment to CXCR1-CXCR6, CCR1-CCR10, XCR1, CX3CR1 or ACKR1-ACKR4, suggesting that none is a functional receptor for CXCL14. As positive control, cognate ligands at 200 nM showed the expected responses, including ACKR2-ACKR4, which are known to scavenge chemokines *via* receptor desensitization and internalization. Identical results were obtained in the β-arrestin-1 recruitment assay (not shown), i.e., 300 nM CXCL14 did not induce a response with any of the listed receptors. Lack of β-arrestin recruitment does not exclude the possibility of CXCL14 binding, which enables CXCL14 to act as positive allosteric modulator for CXCR4 (29). We applied the β-arrestin recruitment assay to the study of CXCL14 synergism. Interestingly, strong synergism was observed with 3 nM CCL19 in combination with increasing concentrations of CXCL14, reaching >70% of maximal β-arrestin responses seen at the highest concentrations of CCL19 when tested alone (Figure 1B). CXCL14 on its own was completely inactive throughout the concentration range tested. These results demonstrate that CXCL14 synergism is not restricted to CXCR4 and its ligand CXCL12.

**FIGURE 2:**
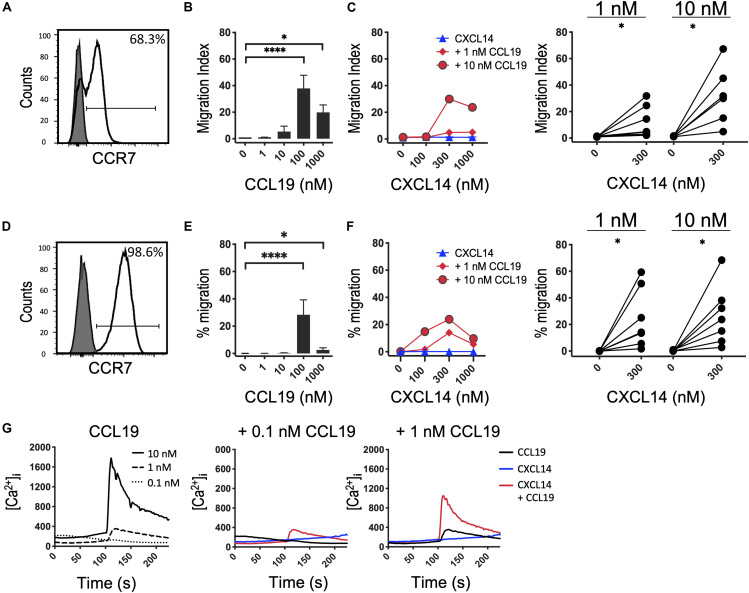
CXCL14 synergizes with the CCR7 ligand CCL19 in the induction of chemotactic and Ca^2+^-mobilization responses. **(A)** Surface expression of CCR7 on primary CD3^+^ T cells (clear histogram), gray histogram indicates fluorescence-minus one control. **(B)** Migration of primary T cells toward CCL19, data shown are means + SEM of 6–7 independent experiments. ^∗^*P* < 0.05 and ^****^P < 0.0001 compared to 0 nM using Friedman test followed by Dunn’s multiple comparisons test. **(C)** Migration of T cells toward CXCL14 in combination with a fixed concentration of CCL19. Left panel shows representative data of 7 independent experiments (right panel). ^∗^*P* < 0.05 using Wilcoxon test. **(D)** Surface expression of CCR7 on 300-19-CCR7 cells. **(E)** Chemotactic migration of 300-19-CCR7 cells toward CCL19. Data shown are means + SEM of 7 independent experiments. ^∗^*P* < 0.05 and ^****^P < 0.0001 compared to 0 nM using Friedman test followed by Dunn’s multiple comparisons test. **(F)** Migration of 300-19-CCR7 cells toward CXCL14 and a fixed concentration of CCL19. Left panel shows representative data of 7 independent experiments (right panel). ^∗^*P* < 0.05 using Wilcoxon test. **(G)** Changes in cytoplasmic free Ca^2+^ concentrations upon addition of various concentrations of CCL19, 300 nM CXCL14 or combinations of 0.1 or 1 nM CCL19 with 300 nM CXCL14. One representative set of measurements from 4 independent experiments is shown.

Next, we decided to further investigate the potential synergism between CXCL14 and CCR7 ligands by studying chemotaxis, the prototypical cellular response to chemokines. CCR7 is expressed on naive and central memory (T_CM_) cells, which together make up approximately two-thirds of all T cells present in PBMC (Figure 2A). CCL19 and CCL21, the two selective chemokines for CCR7, induced typical bell-shaped migration responses in T cells with maximal responses seen at 100 nM of CCL19 (Figure 2B) or CCL21 (Supplementary Figure S1), whereas CXCL14 up to 1,000 nM was completely inactive (Figure 2C). However, CCL19 at two submaximal concentrations, 1 or 10 nM, resulted in strong synergistic responses when combined with 300–1,000 nM CXCL14. Maximal synergism was consistently observed at 300 nM CXCL14 and declined at 1,000 nM CXCL14, resembling bi-modal response curves typically seen with chemokines. To compensate for substantial inter-experimental variations with PBMC from various blood donors, paired values of chemotactic indices for each experiment are shown in a line-plot. More importantly, and with only a single exception, 300 nM CXCL14 consistently induced synergistic cell migration, irrespective of the type or concentration of CCR7 ligand used. These robust synergistic responses were replicated in CCR7-transfected 300-19 pre-B cells (Figures 2D–F). The chemotaxis data generated with 300-19-CCR7 cells were presented as net migration in percentage of total input cells. Again, strong synergistic responses were seen with CXCL14 in combination with 1 or 10 nM CCL19 and maximal responses were consistently obtained in combination with 100–1,000 nM CXCL14. In line with primary T cells from PBMC, CCL19 induced maximal responses at 100 nM and maximal synergistic responses were observed with 300 nM CXCL14 in combination with 1 or 10 nM CCL19. Again, CXCL14 on its own was inactive. These findings were fully replicated with CCL21, the second ligand for CCR7, i.e., strong synergistic responses were again observed with 300 nM CXCL14 in combination with 1 or 10 nM CCL21, both with primary T cells and 300-19-CCR7 cells (Supplementary Figure S1).

Finally, in analogy to our previous study (29), we extended our investigations to Ca^2+^-mobilization, which is a fast and transient signaling event induced upon binding of chemokines to their receptors (38, 39). This highly sensitive assay reveals Ca^2+^ signals at chemokine concentrations well below those needed for cell migration, as demonstrated with CCL19 and CCL21 in 300-19-CCR7 cells (Figure 2G and Supplementary Figure S1E). 300 nM CXCL14 on its own was inactive yet in combination with 0.1 or 1 nM CCL19 or CCL21 generated synergistic Ca^2+^-responses, indicating that CXCL14 synergism extends to an early, G_α i_-type G-protein-mediated signaling event. We conclude that CXCL14 is a highly potent synergistic chemokine in combination with CCL19 or CCL21 and CCR7-expressing target cells.

**FIGURE 3:**
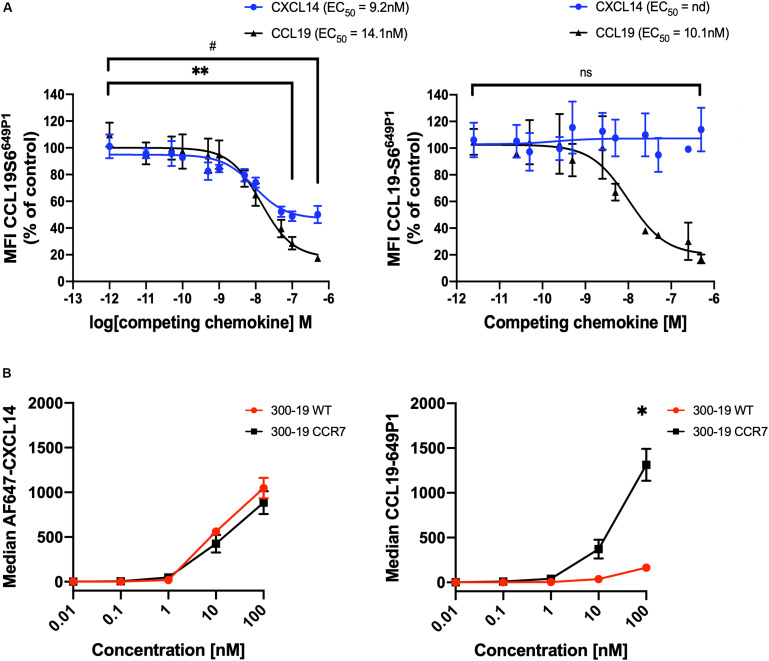
CXCL14 interferes with CCL19 internalization. **(A)** CXCL14 interaction with CCR7 was determined indirectly in competition binding assays with fluorescently labeled CCL19 (CCL19^Dy649P1^) by flow cytometry. 300-19-CCR7 cells were incubated with 25 nM of CCL19^Dy649P1^ in the presence of increasing concentrations of unlabeled human CCL19 or CXCL14 at 37°C (left panel) or 10°C (right panel). Competition is displayed as a decrease in mean fluorescence intensity (non-linear regression applied) of the cell-associated labeled chemokine in comparison to untreated cells. EC_50_, concentration giving half-maximal inhibition. Data shown are means + SEM of 3–4 independent experiments. ^∗∗^*P* < 0.01 comparing CXCL14 addition to untreated cells, ^#^*P* < 0.05 comparing CCL19 addition to untreated cells, using Wilcoxon test. **(B)** Binding of increasing concentrations of AF647-CXCL14 (left panel) and CCL19^Dy649P1^ (right panel) to 300-19-WT or 300-19-CCR7 was determined by flow cytometry. Data shown are means ± SD of 3 independent experiments. ^∗^*P* < 0.05 compared to 0 nM using Holm-Sidak test.

We questioned whether CXCL14 was able to interact with CCR7-expressing cells and, in addition, to interfere with the interaction between CCL19 and CCR7. First, we carried out competition experiments at 37°C with 300-19-CCR7 cells in combination with a fixed concentration of fluorescently labeled CCL19 (CCL19^Dy649P1^) that fully retained its biological activity (32) and increasing concentrations of competing ligands, either unlabeled CCL19 or CXCL14. Parental 300-19 cells lacking CCR7 were used to determine the level of background staining. The data clearly show a significant yet partial reduction in CCL19^Dy649P1^ fluorescence at high CXCL14 concentrations reaching 50 ± 6% at 500 nM CXCL14 (Figure 3A). However, reduction of the incubation temperature to 10°C, which prevents CCR7 internalization, neutralized this effect, suggesting that CXCL14 interfered with receptor internalization rather than CCL19 binding to CCR7. In addition, using 300-19-CCR7 and parental 300-19 cells at 10°C together with Alexafluor-647 labeled CXCL14 (AF647-CXCL14; which retained full biological activity), we show that CXCL14 was unable to bind specifically to CCR7 (Figure 3B). Of note, the observed level of CXCL14 binding to 300-19 was comparable to the level of specific CCL19 binding to CCR7. These findings fully agree with our previous report of AF647-CXCL14 binding to CXCR4 expressed on 300-19 cells, which was undetectable by flow cytometry (29). In this study, specific interaction with CXCR4 was only observed by surface plasmon resonance (SPR) spectroscopy using sensor chips coated with CXCR4-expressing lentivirus-like particles. Unfortunately, lack of corresponding reagents expressing CCR7 prevented us from carrying out CCR7-specific SPR studies. Currently, we conclude that binding of CXCL14 to 300-19 cells reduced CCL19-mediated internalization of CCR7 at physiological temperature.

### CXCL14 Synergism Extends to Additional Homeostatic Chemokine Systems

**FIGURE 4:**
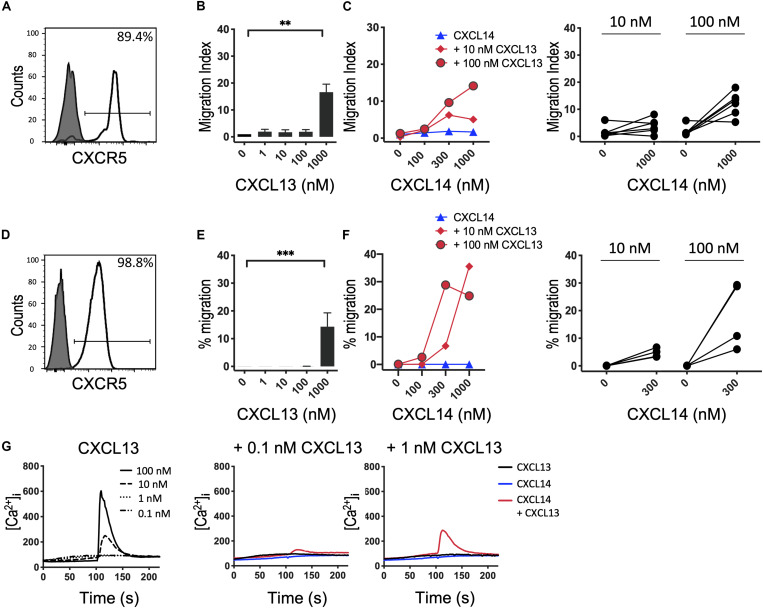
CXCL14 synergizes with the CXCR5 ligand CXCL13 in the induction of chemotactic and Ca^2+^ mobilization responses. **(A)** Surface expression of CXCR5 on primary CD19^+^ B cells (clear histogram), gray histogram indicates fluorescence-minus one control. **(B)** Migration of primary B cells toward CXCL13. Data shown are means + SEM of 6 independent experiments. ^∗∗^*P* < 0.01 compared to 0 nM using Friedman test followed by Dunn’s multiple comparisons test. **(C)** Migration of B cells toward CXCL14 in combination with a fixed concentration of CXCL13. Left panel shows representative data of 6 independent experiments (right panel). **(D)** Surface expression of CXCR5 on 300-19-CXCR5 cells. **(E)** Chemotactic migration of 300-19-CXCR5 cells toward CXCL13. Data shown are means + SEM of 8 independent experiments. ^∗∗∗^*P* < 0.001 compared to 0 nM using Friedman test followed by Dunn’s multiple comparisons test. **(F)** Migration of 300-19-CXCR5 cells toward CXCL14 and a fixed concentration of CXCL13. Left panel shows representative data of 4 independent experiments (right panel). **(G)** Changes in cytoplasmic free Ca^2+^ concentration upon addition of various concentrations of CXCL13, 300 nM CXCL14 or combinations of 0.1 or 1 nM CXCL13 and 300 nM CXCL14. One representative set of measurements out of 4–5 independent experiments is shown.

Based on our results with CXCR4 (29) and CCR7 (shown above), we speculated that the synergism of CXCL14 extends to other chemokine/chemokine receptor systems involved in homeostatic immune processes, such as CXCR5 and its single ligand CXCL13. Indeed, studies with B cells from peripheral blood (Figures 4A–C) and CXCR5-transfected 300-19 cells (Figures 4D–F), which both express uniform and high levels of CXCR5, revealed strong synergism with CXCL14. As previously reported (40), CXCL13 is not a very potent chemokine for CXCR5-expressing cells, showing maximal responses at >100 nM, resembling the low potency of CXCL14 on human blood monocytes (2, 3). CXCL14 on its own was inactive on B cells. Interestingly, and in accordance with the low potency of CXCL13, maximal synergistic responses were frequently observed in combination with the highest CXCL14 concentration (1,000 nM) tested, as opposed to our results with CXCR4 and CCR7 where 300 nM gave maximal synergistic responses. Further, we have noticed that the synergistic responses with primary B cells were relatively low, which may be due to using unfractionated PBMC, containing numerous CXCR4/CCR7-high expressing leukocytes that might have interfered in this assay. This potential problem was circumvented by using 300-19-CXCR5 cells that uniformly express CXCR5 but lack human CXCR4 and CCR7. Of note, 300-19 cells express low levels of endogenous murine CXCR4, but it did not mediate responses to CXCL12. Nevertheless, the representative results in Figure 4 demonstrate a strong synergism between CXCL14 and CXCL13 that clearly exceeded maximal 300-19-CXCR5 cell migration responses to CXCL13 alone (Figure 4F). In addition, Ca^2+^-mobilization curves were readily detected at low CXCL13 concentrations, again well below those required for induction of cell migration. As expected, 300 nM CXCL14 alone was inactive, yet induced small but detectable responses in combination with 0.1 and 1 nM CXCL13 (Figure 4G). We conclude that, indeed, the potent synergistic function of CXCL14 includes all major constitutive lymphoid tissue-homing chemokine systems.

### CXCL14 Shows Weak Interactions With Inflammatory Chemokine Systems

**FIGURE 5:**
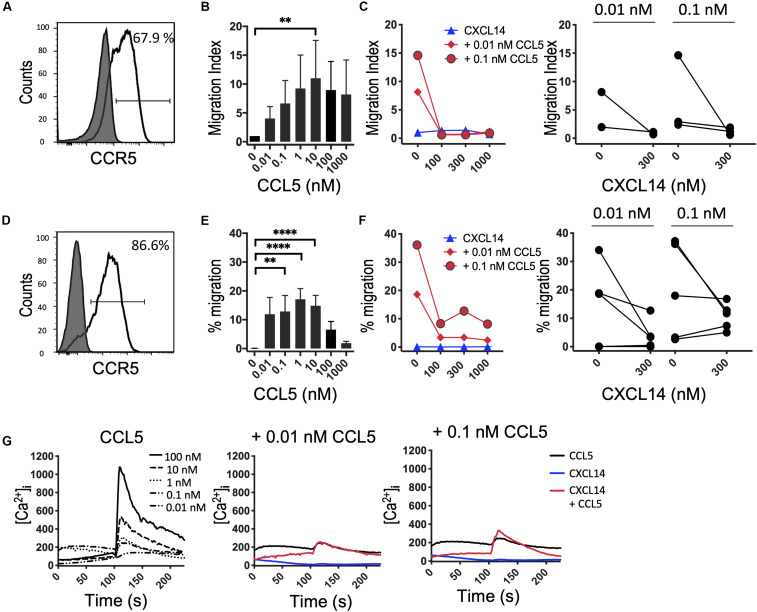
CXCL14 does not synergize with the CCR5 ligand CCL5 in chemotaxis. **(A)** Surface expression of CCR5 on primary human CD3^+^ T cells cultured with IL-2 and IL-15 (clear histogram), gray histogram indicates fluorescence-minus one control. **(B)** Migration of primary T cells toward CCL5. Data shown are means + SEM of 3 independent experiments. **(C)** Migration of T cells toward CXCL14 in combination with CCL5. Left panel shows representative data of 2–3 experiments (right panel). **(D)** CCR5 cell surface expression on 300-19-CCR5 cells (clear histogram). **(E)** Chemotactic migration of 300-19-CCR5 cells toward CCL5. Data shown are means + SEM of 8 independent experiments. **(F)** Migration of 300-19-CCR5 cells toward CXCL14 in combination with CCL5. Left panel shows representative data of 5–6 independent experiments (right panel). ^∗∗^*P* < 0.01 and ^****^P < 0.0001 compared to 0 nM using Friedman test followed by Dunn’s multiple comparisons test. **(G)** Changes in cytoplasmic free Ca^2+^ upon addition of various concentrations of CCL5, 300 nM CXCL14 or combinations of 0.01 or 0.1 nM CCL5 and 300 nM CXCL14. One set of measurements from 3 independent experiments is shown.

It is possible that the CXCL14 synergism is selective for homeostatic chemokine systems, implying that similar synergistic responses would not be seen with inflammatory chemokines. An initial indication that this may be true stems from our earlier studies demonstrating that CXCL14 was unable to synergize with CCL2 and its receptor CCR2, an inflammatory chemokine system involved in the recruitment of myeloid cells, including blood monocytes, to inflamed tissues (29). Here, we extended these studies to inflammatory chemokine systems controlling the recruitment of effector T cells. First, we turned to CCL5 and one of its receptors CCR5 that is prominently expressed on activated Th1 cells (41). In this system we were unable to detect obvious and consistent CXCL14-mediated synergism (Figure 5). CCR5 is expressed on a minor subset of T cells present in peripheral blood and, therefore, we did not use fresh PBMC in our chemotaxis assay. Instead, we used expanded T cell lines derived from primary T cells that were stimulated with anti-CD3/CD28 beads and then cultured for 14–21 days in the presence of IL-2 and IL-15. The majority of expanded T cells expressed CCR5 (Figure 5A) and CXCR3 (see below) as well as various combinations of other receptors for inflammatory chemokines. In addition, to circumvent the potential problem posed by the co-expression of multiple chemokine receptors, we also used 300-19-CCR5 cells, showing uniform and selective expression of CCR5 (Figure 5D). Both readily migrated in response to CCL5 with maximal migration seen at <100 nM whereas CXCL14 on its own was inactive. Interestingly, as opposed to synergism, addition of CXCL14 to suboptimal concentrations (0.01 and 0.1 nM) of CCL5 inhibited chemotactic migration. Statistical significance was not reached yet these observations were consistently made with both primary and CCR5-transfected cell lines. Although we do not know the underlying mechanism at present, inter-experimental variation can be excluded since inhibition was clearly evident in 4 out of 6 experiments at both concentrations of CCL5 (Figure 5F). By contrast to inhibition of chemotaxis, modest synergistic Ca^2+^ spikes were measured as evidenced in the example depicted in Figure 5G. CCL5 is a highly potent agonist for induction of Ca^2+^ changes in 300-19-CCR5 cells and small responses were already detected at CCL5 concentrations as low as 0.01 nM whereas, CXCL14 on its own was inactive. We conclude that CXCL14 does not synergize with CCL5 and its receptor CCR5. The reported inhibitory effect was not further examined but points to some sort of interaction between CXCL14 and CCR5-expressing cells that appears to be unrelated to the cellular background (human T cell cultures vs. murine pre-B cell line).

**FIGURE 6:**
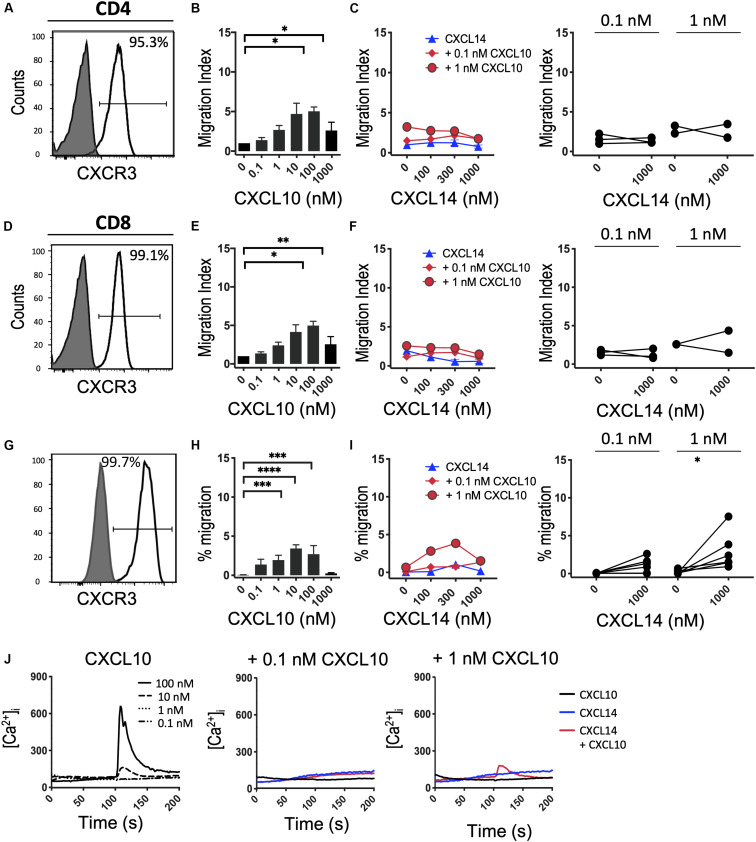
CXCL14 does not show consistent synergism with the CXCR3 ligand CXCL10. **(A,D)** Surface expression of CXCR3 on primary CD4^+^ or CD8^+^ cells cultured with IL-2 and IL-15 (clear histogram), gray histogram indicates staining with fluorescence-minus one control. **(B,E)** Migration of primary CD4^+^ or CD8^+^ cells toward CXCL10. Data shown are means + SEM of 4 independent experiments. **(C,F)** Migration of primary CD4^+^ or CD8^+^ cells toward CXCL14 and a fixed concentration of CXCL10. Left panel shows representative data of 2–3 experiments (right panel). **(G)** CXCR3 cell surface expression on 300-19-CXCR3 cells (clear histogram). **(H)** Chemotactic migration of 300-19-CXCR3 cells toward CXCL10. Data shown are means + SEM of 12 independent experiments. ^∗^*P* < 0.05 and ^∗∗^*P* < 0.01 ^∗∗∗^*P* < 0.001 and ^****^P < 0.0001 compared to 0 nM of the indicated chemokine using Friedman test followed by Dunn’s multiple comparisons test. **(I)** Migration of 300-19-CXCR3 cells toward CXCL14 and a fixed concentration of CXCL10. Left panel shows representative data of 6 experiments (right panel). ^∗^*P* < 0.05 using Wilcoxon test. **(J)** Changes in cytoplasmic free Ca^2+^ upon addition of various concentrations of CXCL10, 300 nM CXCL14 or combination of CXCL10 and 300 nM CXCL14. One representative set of measurements from 3–4 independent experiments is shown.

Finally, we turned to CXCR3, which, similarly to CCR5, is upregulated in activated T cells under tissue culture conditions in the presence of IL-2 or IL-2 plus IL-15 (42). *In vivo*, CXCR3 recruits Th1 cells to inflammatory sites dominated by IFNγ and other pro-inflammatory cytokines (which induce the CXCR3-specific chemokines). Consistent with the increased promiscuity of the inflammatory chemokine receptors for chemokines, CXCR3 is selectively activated by three related chemokines, CXCL9, CXCL10 and CXCL11. Figures 6A–F shows that expanded CD4^+^ and CD8^+^ T cells uniformly expressed this receptor and efficiently migrated to CXCL10 with peak responses seen at 100 nM. As reported in Figure 5, CXCL14 by itself was inactive. Also, CXCL14 failed to consistently synergize with CXCL10 and, by contrast to CCL5 (Figure 5), an inhibitory effect was not observed. For reasons unknown at present, a weak synergism between CXCL14 and CXCL10 was detected when 300-19-CXCR3 cells were used but this effect was restricted to 1 nM CXCL10 in combination with 300 nM CXCL14 (Figures 6H,I). The above results were replicated with CXCL11, including an unexplained synergism with 300-19-CXCR3 cells between 1 nM CXCL11 and 300 nM CXCL14 (Supplementary Figure S2). As shown in Figure 6J, prominent Ca^2+^ changes were not readily detected in 300-19-CXCR3 cells when “sub-active” concentrations of CXCL10 were combined with 300 nM CXCL14. Occasional (inconsistent) Ca^2+^ spikes may reflect the superior sensitivity of this assay as compared to chemotactic migration. In summary, and based on our previous study of CCR2 (29) and our current study of CCR5 and CXCR3 and their respective chemokine ligands, we conclude that CXCL14 does not synergize with inflammatory chemokine systems as prominently (and consistently) as seen in homeostatic chemokine systems.

## Discussion

We here demonstrate that the highly potent synergistic effect of CXCL14 with other chemokines goes well beyond CXCL12 as published recently (29). The most robust and reproducible synergistic responses were seen during *in vitro* chemotaxis assays, the prototypical test for defining the functionality and target cell specificity of chemokines. Based on these results we conclude that CXCL14 preferentially synergizes with the major homeostatic chemokines involved in controlling steady-state processes as diverse as immune surveillance of secondary lymphoid tissues (CXCL13, CCL19, CCL21) and tissue development (CXCL12) (42, 43). Migration responses of CXCL14 in combination with inflammatory chemokines at sub-active concentrations, i.e., concentrations below the threshold for induction of chemotaxis, did not reveal clear-cut and consistent synergistic responses. CXCR3-mediated responses with 300-19-CXCR3 cells represent a notable exception, where we have observed substantial and as yet unexplained inter-experimental variations over a time period of several years. Without excluding “outlier” results, CXCL14 synergized with both CXCL10 and CXCL11 at sub-optimal (1 nM) concentrations. The outcome of *in vitro* chemotaxis is influenced by several factors, including differences in the running time of the assay and the type and quality of target cells, posing significant problems for the statistical analysis of combined data collected from numerous individual experiments. Perhaps it is more prudent to show all results individually, in which case the CXCL10 and CXCL11 cannot be considered synergistic partners for CXCL14 (Figure 6 and Supplementary Figure S2). In contrast to *in vitro* chemotaxis, Ca^2+^ mobilization responses are much shorter in terms of initiation (<10 s following addition of chemokines) and duration (seconds), which may explain their increased sensitivity to low concentrations of chemokines. Our Ca^2+^ mobilization experiments confirmed the synergism seen between CXCL14 and homeostatic chemokines CXCL13, CCL19, and CCL21 during *in vitro* chemotaxis and agrees with our previous CXCL12 studies (29). Interestingly, small synergistic Ca^2+^ signals were occasionally observed with CXCL14 in combination with the inflammatory chemokines (CXCL10, CXCL11, CCL5) whereas CXCL14 at concentrations of up to 1 μM was inactive on its own, irrespective of the type of cells examined (fresh or cultured lymphocytes isolated from peripheral blood or transfected murine pre-B cells expressing the relevant chemokine receptors) or the type of *in vitro* assay (chemotaxis, Ca^2+^ mobilization, β-arrestin recruitment) employed. An exhaustive examination of synergism between CXCL14 and all known chemokines was well beyond the scope of the present study. Still, in a next step, we propose to turn our attention to those chemokines that are known to control immune cell traffic in peripheral tissues, i.e., chemokines known to be co-expressed with CXCL14 under steady-state conditions in peripheral tissues, including skin (CCL1/CCR8) and gut (CCL25/CCR9).

Diverse mechanisms have been shown to underlie chemokine synergy, which collectively translate to enhanced immune cell migration (42, 43). Of these, the most prominent ones involve the formation of chemokine heterocomplexes (44). It is thought that the physical interactions between two synergizing chemokines results in favorable conformational changes that lower the threshold concentration of one of the complexed chemokines for activation of cells expressing the corresponding chemokine receptor. Heterocomplex-based synergism is not restricted to chemokines, in that proteins other than chemokines are able to complex with chemokines and improve their activity (45–50). In addition to chemokine heterocomplex formation, sequential and/or simultaneous triggering of more than one type of chemokine receptor has also been shown to result in synergistic outcomes in target cells (51). The underlying mechanisms for this observation was proposed to be due to chemokine receptor-mediated intersecting signaling pathways leading to synergistic as opposed to additive cell responses. This is best exemplified by an earlier study using cells co-expressing CXCR4 and CCR5 in combination with selective receptor antagonists (AMD3100 and maraviroc, respectively) (52). Finally, similar to chemokines in solution, chemokine receptors themselves are known to form dynamic aggregates through lateral movements on cell surfaces, which are thought to modulate ligand selectivity and transmembrane signaling properties (53). Chemokine receptor-mediated synergism has been difficult to prove, not least because of the diversity of GPCRs present on immune cells. However, we believe that our previous study of synergism between CXCL14 and CXCL12 made a strong case in favor of this model (29). Here, while interaction between CXCL14 and CXCL12 was ruled out by several methods, CXCL14 triggered CXCR4 clustering as a direct consequence of CXCL14 binding to cell surface CXCR4 (29, 54). In our model, interaction of CXCL14 with CXCR4 causes enhanced receptor oligomerization comprising of conformational intermediates of CXCR4 with reduced activation thresholds that allow signal transduction at low (otherwise sub-active) concentrations of CXCL12. Our current data led us to propose that the synergistic effect of CXCL14 is not restricted to CXCR4 but also includes the homeostatic chemokine receptors CXCR5 and CCR7. The underlying molecular mechanism may involve positive allosteric receptor modulation, although we were unable to demonstrate direct binding of CXCL14 to CCR7 or CXCR5. Since CXCL14 strongly interacts with proteoglycans (29, 55), it is possible that CXCL14 targets carbohydrate modifications on cell surface proteins, including CCR7. Also, SPR spectroscopy monitors molecular interactions in real-time, which may be more sensitive than steady-state receptor binding protocols (e.g., flow cytometry) and, thus, explain our previous results with CXCR4. Nevertheless, the substantial functional synergism of CXCL14 with chemokines for CXCR4, CXCR5 and CCR7 provides strong impetus to further investigate this phenomenon at the molecular level.

The physiological relevance of our findings is not clear at present. In fact, *in vivo* models for the study of chemokine synergy are rare. Evidence in support of chemokine synergism was provided by studies involving the combinatorial injections of CXCL10 and CCL5 (56), CXCL6 and CCL7 (57) or CXCL1, CXCL2 and CXCL3 (58), leading to more than additive numbers of recruited immune cells at sites of injection. Combination of CXCL4 and CCL5 was shown to exacerbate disease in a mouse model of atherosclerosis (59), and CXCL12 in combination with HMGB1 enhanced monocyte recruitment in a mouse model of tissue damage (49). In our own preliminary studies, we failed to detect a significant synergistic recruitment of immune cells in response to intraperitoneal administration of CXCL14 and CXCL12 (unpublished observations). Nevertheless, the observed strong synergy with homeostatic chemokines suggests that synergistic effects involving CXCL14 play a role in healthy, rather than inflamed, tissues at sites where these chemokines are co-expressed under constitutive conditions. In fact, we know that CXCL14 is down-regulated under inflammatory conditions (2, 7), ruling out a primary influence on the recruitment of short-lived effector leukocytes. Of interest, CXCL14 and CXCL12 are the most highly conserved members of the large family of chemokines and mice lacking these chemokines have severe developmental defects [reviewed in McCully et al. and Nagasawa (1, 60)]. It is possible that CXCL14 and CXCL12 synergize during organ development, including neurovascular patterning (61), eye (62) and connective tissue (63) formation, where both chemokines are prominently expressed. Of interest, both chemokines were shown to activate fibroblasts, suggesting that CXCL14 and CXCL12 may co-operate in controlling fibroblast functions in various scenarios, including tissue repair, fibrosis (23–25) and tumor progression (27, 28, 64). CXCL14/CXCL12 synergism could also play a role in metabolic processes, including glucose metabolism (20) and thermoregulation (65) where lack of CXCL14 was associated with impaired macrophage recruitment and abnormal brown adipose tissue formation. Besides CXCL12, we here show that CXCL14 synergizes with CXCL13, CCL19 and CCL21, homeostatic chemokines fulfilling critical functions in the control of primary T and B cell responses (43). Their discrete expression contributes to the micro-anatomical segregation of T and B cell zones within secondary lymphoid tissues and disease-associated ectopic lymphoid-like structures (43, 66). There is no evidence of CXCL14 production at these sites, but tissue-derived CXCL14 may gain access *via* the lymphatic system. Verification of these models awaits the generation of mouse models featuring conditional CXCL14 expression at discrete tissue locations.

In conclusion, the present study demonstrates that synergy of CXCL14 is not restricted to CXCL12 but instead extends to the homeostatic chemokines CXCL13, CCL19 and CCL21 and their cognate receptors. Consistent synergistic effects with inflammatory chemokines CXCL10, CXCL11, CCL2 (29) and CCL5 were not detected. It is possible that the evolutionary conservation of CXCL14 is associated with fine-tuning multiple local immune and/or tissue cell responses in co-operation with homeostatic chemokines in peripheral tissues where CXCL14 is abundantly expressed.

## Data Availability Statement

The raw data supporting the conclusions of this article will be made available by the authors, without undue reservation.

## Ethics Statement

The studies involving human participants were reviewed and approved by the Wales Research Ethics Committee (ethics approval number: 08/WSE04/17). The patients/participants provided their written informed consent to participate in this study.

## Author Contributions

AK, PC, DL, SS, PP, AC, ME, and BM designed research. AK, PC, MiM, MaM, MA, and MS performed research and analyzed data. AK, PC, DL, SS, PP, AC, ME, and BM wrote the paper. All authors contributed to the manuscript and approved the submitted version.

## Conflict of Interest

The authors declare that the research was conducted in the absence of any commercial or financial relationships that could be construed as a potential conflict of interest.

## Supplementary Material

Supplementary Figure 1 CXCL14 synergizes with the CCR7 ligand CCL21 in the induction of chemotactic and Ca^2+^-mobilization responses. **(A)** Migration of primary T cells toward CCL21, data shown are means + SEM of 8 independent experiments. ^∗∗^*P* < 0.01 and ^****^*P* < 0.0001 compared to 0 nM using Friedman test followed by Dunn’s multiple comparisons test. **(B)** Migration of T cells toward CXCL14 in combination with a fixed concentration of CCL21. Left panel shows representative data of 7–8 independent experiments (right panel). ^∗^*P* < 0.05, ^∗^*P* < 0.01 using Wilcoxon test. **(C)** Chemotactic migration of 300-19-CCR7 cells toward CCL21. Data shown are means + SEM of 8 independent experiments. **(D)** Migration of 300-19-CCR7 cells toward CXCL14 and a fixed concentration of CCL21. Left panel shows representative data of 7 independent experiments (right panel). **(E)** Changes in cytoplasmic free Ca^2+^ concentrations upon addition of various concentrations of CCL21, 300 nM CXCL14 or combinations of 0.1 or 1 nM CCL21 with 300 nM CXCL14. One representative set of measurements from 4 independent experiments is shown.

Supplementary Figure 2 CXCL14 does not show consistent synergism with the CXCR3 ligand CXCL11. **(A)** Chemotactic migration of 300-19-CXCR3 cells toward CXCL11. Data shown are means + SEM of 4 independent experiments. ^∗^*P* < 0.05 and ^∗∗^*P* < 0.01 compared to 0 nM using Friedman test followed by Dunn’s multiple comparisons test. **(B)** Migration of 300-19-CXCR3 cells toward CXCL14 and a fixed concentration of CXCL11. Left panel shows representative data of 3–4 independent experiments (right panel). **(C)** Changes in cytoplasmic free Ca^2+^ concentrations upon addition of various concentrations of CXCL11, 300 nM CXCL14 or combinations of 0.1 or 1 nM CXCL11 with 300 nM CXCL14. One representative set of measurements from 3 independent experiments is shown.
