# Supplementary figures and images for: CXCL14 Preferentially Synergizes With Homeostatic Chemokine Receptor Systems
Source: Front Immunol. 2020 Oct 5;11:561404. doi: 10.3389/fimmu.2020.561404 (PMC7570948; doi:10.3389/fimmu.2020.561404)

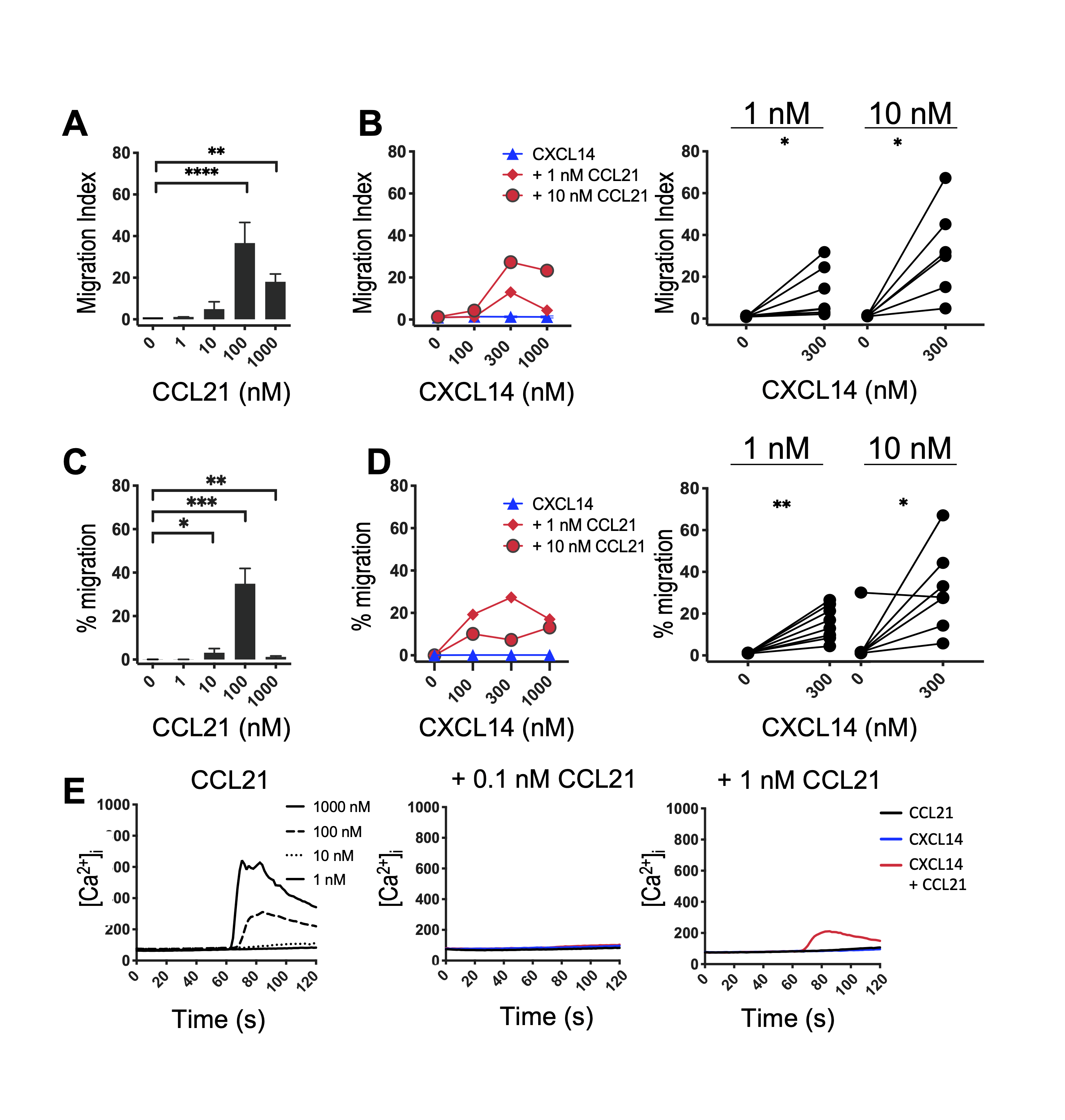

Supplement: Supplementary Figure 1 — CXCL14 synergizes with the CCR7 ligand CCL21 in the induction of chemotactic and Ca2+-mobilization responses. (A) Migration of primary T cells toward CCL21, data shown are means + SEM of 8 independent experiments. ∗∗P < 0.01 and ****P < 0.0001 compared to 0 nM using Friedman test followed by Dunn’s multiple comparisons test. (B) Migration of T cells toward CXCL14 in combination with a fixed concentration of CCL21. Left panel shows representative data of 7–8 independent experiments (right panel). ∗P < 0.05, ∗P < 0.01 using Wilcoxon test. (C) Chemotactic migration of 300-19-CCR7 cells toward CCL21. Data shown are means + SEM of 8 independent experiments. (D) Migration of 300-19-CCR7 cells toward CXCL14 and a fixed concentration of CCL21. Left panel shows representative data of 7 independent experiments (right panel). (E) Changes in cytoplasmic free Ca2+ concentrations upon addition of various concentrations of CCL21, 300 nM CXCL14 or combinations of 0.1 or 1 nM CCL21 with 300 nM CXCL14. One representative set of measurements from 4 independent experiments is shown. [file Image_1.tiff]

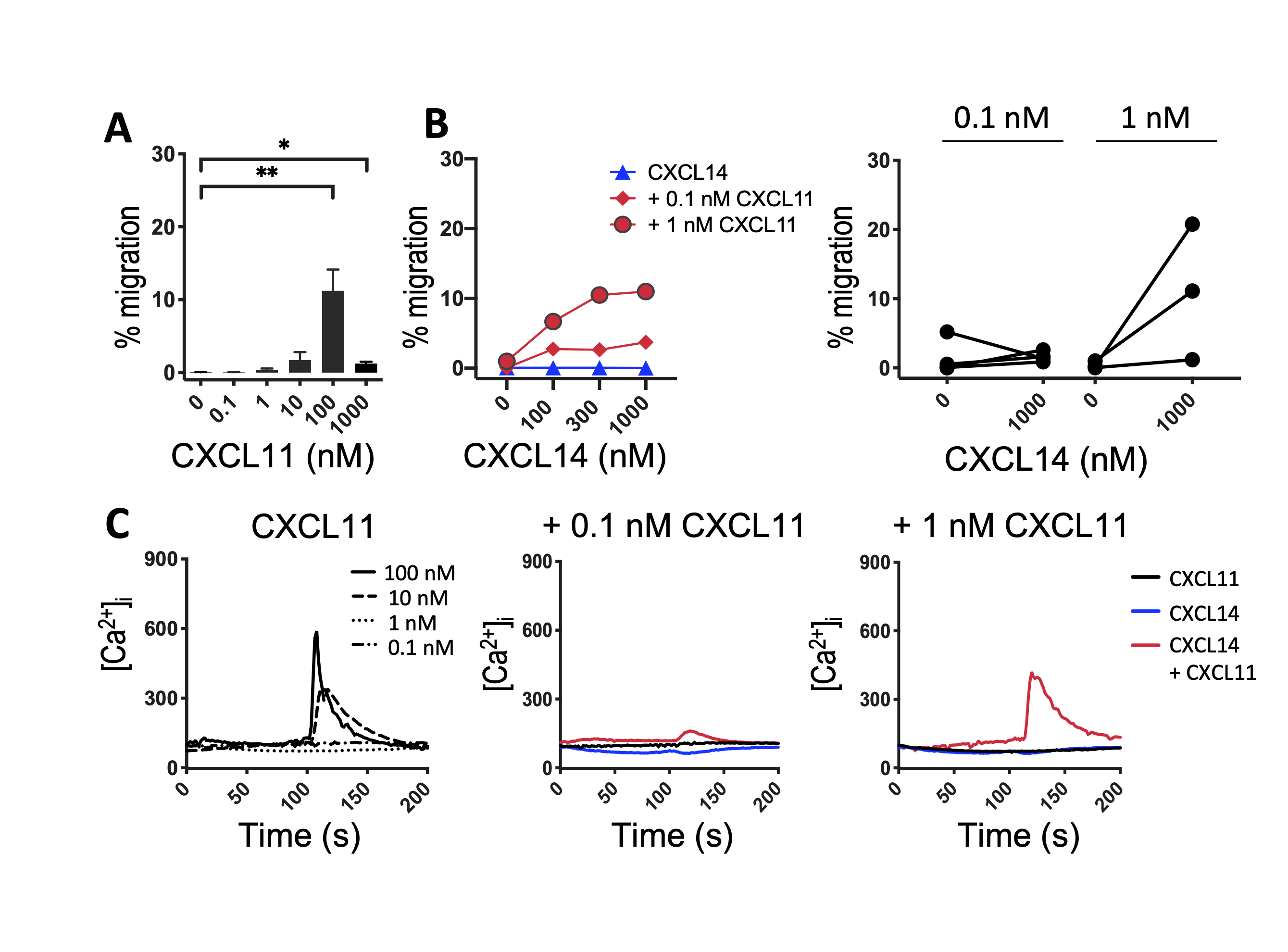

Supplement: Supplementary Figure 2 — CXCL14 does not show consistent synergism with the CXCR3 ligand CXCL11. (A) Chemotactic migration of 300-19-CXCR3 cells toward CXCL11. Data shown are means + SEM of 4 independent experiments. ∗P < 0.05 and ∗∗P < 0.01 compared to 0 nM using Friedman test followed by Dunn’s multiple comparisons test. (B) Migration of 300-19-CXCR3 cells toward CXCL14 and a fixed concentration of CXCL11. Left panel shows representative data of 3–4 independent experiments (right panel). (C) Changes in cytoplasmic free Ca2+ concentrations upon addition of various concentrations of CXCL11, 300 nM CXCL14 or combinations of 0.1 or 1 nM CXCL11 with 300 nM CXCL14. One representative set of measurements from 3 independent experiments is shown. [file Image_2.tiff]
